# New insights into the kinetics of bacterial growth and decay in pig manure–wheat straw aerobic composting based on an optimized PMA–qPCR method

**DOI:** 10.1111/1751-7915.13380

**Published:** 2019-03-05

**Authors:** Jinyi Ge, Guangqun Huang, Xiaoxi Sun, Hongjie Yin, Lujia Han

**Affiliations:** ^1^ Biomass Resources and Utilization Laboratory College of Engineering China Agricultural University (East Campus) Beijing 100083 China; ^2^ Department of Civil and Environmental Engineering Princeton University Princeton NJ 08540 USA

## Abstract

Aerobic composting is a bacteria‐driven process to degrade and recycle wastes. This study quantified the kinetics of bacterial growth and decay during pig manure–wheat straw composting, which may provide insights into microbial reaction mechanisms and composting operations. First, a propidium monoazide–quantitative polymerase chain reaction (PMA–qPCR) method was developed to quantify the viable bacteria concentration of composting samples. The optimal PMA concentration and light exposure time were 100 μM and 8 min respectively. Subsequently, the concentrations of total and decayed bacteria were quantified. Viable and decayed bacteria coexisted during the entire composting period (experiments A and B), and the proportion of viable bacteria finally fell to only 35.1%. At the beginning, bacteria grew logarithmically and decayed rapidly. Later, the bacterial growth in experiment A remained stable, while that of experiment B was stable at first and then decomposed. The duration of the stable stage was positively related to the soluble sugar content of composting materials. The logarithmic growth and rapid decay of bacteria followed Monod equations with a specific growth (0.0317 ± 0.0033 h^−1^) and decay rate (0.0019 ± 0.0000 h^−1^). The findings better identified the bacterial growth stages and might enable better prediction of composting temperatures and the degree of maturation.

## Introduction

Evolving environmental pollution caused by livestock manure has drawn considerable attention in recent years. As the annual discharge of livestock manure in China currently represents approximately 3.8 billion tons (Wang and Zhang, 2016), improved manure management is both important and necessary. Aerobic composting represents a potentially effective solution capable of tackling these issues owing to its ability to recycle livestock manure as well as other biodegradable wastes into sustainable energy such as organic fertilizers and renewable materials (Haug, 1993).

In essence, aerobic composting is a bacteria‐driven process (Haug, 1993). During composting, bacteria consume organic matter and oxygen to conduct their metabolism, and since this reaction is exothermic, energy is released, which heats up composting materials and pasteurizes most pathogens which are present (Haug, 1993). Knowledge of the kinetics of bacterial growth and decay is crucial to the construction of mechanical models and optimization of operation strategies of composting processes because bacterial metabolism is directly correlated with the variation of composting temperature and degradation of organic matter (Haug, 1993; Mason, 2006). Previous research has employed Monod kinetics to describe bacterial growth and decay during aerobic composting as follows (Mason, 2006; Sole‐Mauri *et al*., 2007):

(1) $$dX_{\mathrm{viable}}/dt=\left(\mu -k_{d}\right)X_{\mathrm{viable}}$$

(2) $$dX_{\mathrm{decay}}/dt=\left(k_{d}X_{\mathrm{viable}}\right)$$

where *X*_viable_ is the viable bacteria concentration; *t* is the composting time; *μ* is the specific growth rate coefficient; *k*_d_ is the decay rate coefficient; *X*_decay_ is the decayed bacteria concentration.

Scientists have applied different methods to determine the values of *μ* and *k*_d_ of composting processes. Quantitative polymerase chain reaction (qPCR) technology was used to quantify the total bacteria concentration (*X*_total_), i.e., the sum of *X*_viable_ and *X*_decay_ (Li *et al*., 2013; Wang *et al*., 2014; Lü *et al*., 2015; Meng *et al*., 2016a,2016b). However, *μ* and *k*_d_ still cannot be calculated according to Eqs. (1) and (2), because there is no method available to characterize *X*_viable_ for composting materials. For this reason, researchers relied upon assumption and empirical regression to calculate estimates of these two parameters. For example, some research assigned *μ* and *k*_d_ by assumption for the composting processes of corncobs, cattle manure–municipal sewage sludge and swine–straw (Stombaugh and Nokes, 1996; Sole‐Mauri *et al*., 2007; Wang and Witarsa, 2016), while others inferred the variation of *X*_viable_ and the values of *μ* and *k*_d_ based on the regression relationship between bacterial growth and the composting temperature and degradation of organic matter (Mason, 2006; Yamada and Kawase, 2006; Vasiliadou *et al*., 2015; Ge *et al*., 2016a,2016b). Not only are *μ* and *k*_d_ the key parameters in interpreting the mechanisms of bacterial growth and decay, but the values also affect the accuracy of numerical simulation of composting processes (Sole‐Mauri *et al*., 2007; Zhang *et al*., 2012; Ge *et al*., 2015; Vasiliadou *et al*., 2015). Consequently, development of a characterization method of *X*_viable_ for composting materials and illustration of the kinetics of bacterial growth and decay during composting are of considerable importance.

Propidium monoazide (PMA) combined with qPCR has been proven to be able to characterize *X*_viable_ (Reyneke *et al*., 2017). The principle is that PMA as a nucleic acid dye binds the exposed DNA of decayed cells by photoactivation and thereby inhibits qPCRs in decayed bacteria. With the decayed bacteria thus disabled, in a sense, and prevented from contributing their amplified DNA to the result of the qPCR, the resulting amplified amount of bacterial DNA will come from viable cells only (Nocker *et al*., 2006) and can be used to measure the concentration of these viable bacteria, i.e., *X*_viable_. Particularly, Nocker *et al*. (2007) applied the PMA–qPCR method to the detection of viable and decayed bacteria in pure strains, municipal wastewater, estuarine benthic mud and marine sediment. The PMA concentration, light exposure time and light source used in this study were 50 μM, 2 min and a halogen lamp of 650 W. The results showed that these PMA‐treatment conditions were efficient for pure strains but needed to be modified for the mud and sludge samples. Moreover, the study showed that when the PMA concentration was higher than 150 μM, changes in the concentration did not affect the quantitative results. Yu *et al*. (2015) optimized the PMA–qPCR method for chicken samples, using a PMA concentration of 15 μM and a light exposure time of 5 min with a halogen lamp of 650 W. Their results showed that when the PMA concentration was excessively high, it would exert toxic effects on viable bacteria, and they also concluded that the light exposure time should be extended for solid samples. PMA–qPCR has been applied to the detection of viable microbes in a number of previous studies which examined a variety of different samples, and these studies may provide some guidance in the choice of appropriate PMA concentration and light exposure time to be used in such experiments. The PMA‐treatment conditions used in these studies are listed in Table 1. Among the studies of liquid samples (Li *et al*., 2014; Moreno *et al*., 2015; Bonetta *et al*., 2017; Casanovas‐Massana *et al*., 2018; Lu *et al*., 2018), there is little consensus on optimal PMA concentrations, which range from 5 to 100 μM, or on light exposure time, which ranges from 4 to 60 min. In studies of pure strains (Lee *et al*., 2015; Yu *et al*., 2015; Lai *et al*., 2017; Reyneke *et al*., 2017), the PMA concentrations ranged widely, from 15 to 100 μM, but the light exposure time used in all the studies was <5 min. In studies of solid samples (Moreno *et al*., 2015; Cancino‐Faure *et al*., 2016; Youn *et al*., 2017; Casanovas‐Massana *et al*., 2018), the optimal PMA concentration was at a relatively high level, with a range of 50–100 μM; the maximum PMA concentration was 200 μM; and the light exposure time was relatively long, with a range of 8–20 min. In the one study of gas samples (Chang *et al*., 2017), which studied air, the optimal PMA concentration was only 1.5 μM, the lowest among all the studies and all sample types. However, as the technical parameters of PMA treatment differ significantly among different samples, it is necessary to customize the method to suit the characteristics of the specific materials that are being studied (Nocker *et al*., 2007; Yu *et al*., 2015). However, as the technical parameters of PMA treatment differ significantly between various samples, it is necessary to customize the method for specific materials (Nocker *et al*., 2007; Yu *et al*., 2015). Notably, to our best knowledge, there is no report of the application of PMA–qPCR to composting materials.

**Table 1:** PMA‐treatment conditions in previous studies

Material	Optimal PMA concentration	Maximum PMA concentration	Light exposure time	References
Liquid				
Sputum	100 μM	400 μM	20 min	Lu *et al*. (2018)
Spring water	5 μM	/	60 min	Casanovas‐Massana *et al*. (2018)
Water	25 μM	/	5 min	Bonetta *et al*. (2017)
Industry water	50 μM	/	15 min	Moreno *et al*. (2015)
Wastewater	100 μM	/	4 min	Li *et al*. (2014)
Pure strain				
*Lactobacillus gasseri* and *Lactobacillus salivarius*	100 μM	/	5 min	Lai *et al*. (2017)
*Mycobacterium fortuitum*	30 μM	/	2 min	Lee *et al*. (2015)
*Salmonella*	15 μM	40 μM	5 min	Yu *et al*. (2015)
*Legionella Pneumophila*, *Pseudomonas aeruginosa*, *Salmonella typhimurium*, *Staphylococcus aureus* and *Enterococcus faecalis*	50 μM	/	5 min	Reyneke *et al*. (2017)
Solid				
Pig manure–wheat straw	100 μM	200 μM	8 min	This study
Soil	100 μM	/	15 min	Casanovas‐Massana *et al*. (2018)
Chicken	100 μM	/	20 min	Youn *et al*. (2017)
Parasite	100 μM	200 μM	15 min	Cancino‐Faure *et al*. (2016)
Vegetable and shellfish	50 μM	/	15 min	Moreno *et al*. (2015)
Gas				
Air	1.5 μM	/	20 min	Chang *et al*. (2017)

Therefore, the present study was designed to (i) develop a PMA–qPCR method for swine manure–wheat straw composting materials; (ii) quantify the variation of *X*_viable_ during swine manure–wheat straw aerobic composting based on the PMA–qPCR method, determine the variation of *X*_total_ based on qPCR and calculate *X*decay from the results of *X*_viable_ and *X*_total_; and (iii) characterize the values of *μ* and *k*_d_ based on the variation of *X*_viable_ and *X*_decay_. These efforts may help to elucidate the mechanisms of bacterial reactions during composting and provide methodological and data support for improving kinetic models for manure composting.

## Results and discussion

### Pig manure–wheat straw aerobic composting

**Figure 1:**
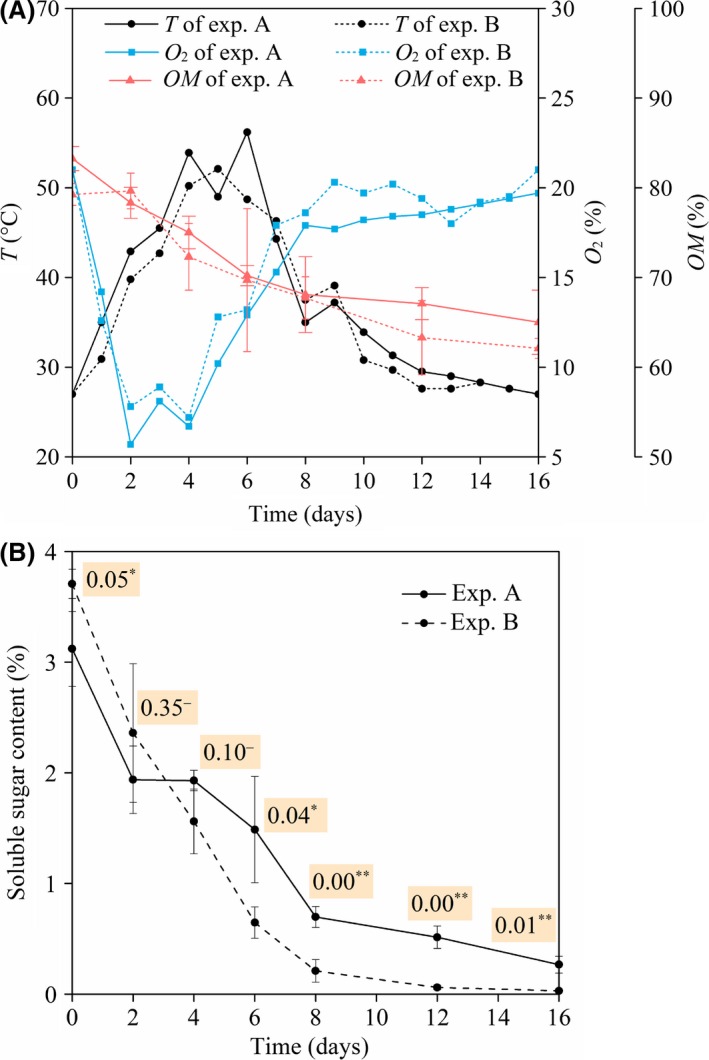
Evolution of (A) *T*, O_2_ and OM and (B) soluble sugar content during pig manure–wheat straw aerobic composting. *Note*:* T* is the composting temperature; O_2_ is the oxygen concentration in the upper part of the composting reactor; OM is the organic matter content; exp represents experiment; the measurements of the OM and soluble sugar content were based on dry weight; error bars of the OM and soluble sugar content represent the standard deviation of triplicate measurements; in Fig. 1B, the numbers followed by ^–^, * or ** represent the significance level (*P*) of the difference between experiments A and B; ^–^ represents no significant difference; * represents significant difference at 0.01 < *P *≤* *0.05; ** represents significant difference at *P *≤* *0.01.

The evolution of temperature, oxygen concentration, organic matter content and soluble sugar content during pig manure–wheat straw aerobic composting is given in Fig. 1. The composting temperature of both experiments A and B experienced mesophilic (days 0–4), thermophilic (days 4–6), cooling (days 6–12) and maturation (days 12–16) phases. During the mesophilic phase, oxygen of both experiments was consumed quickly. The oxygen concentration decreased to minimum and then kept at a relatively low level. During the thermophilic phase, the oxygen concentration increased significantly on day 5, indicating the metabolism of aerobic microbes such as bacteria began to decline. During the cooling–maturation phase, the oxygen concentration of experiment A increased remarkably and then slightly, while the oxygen concentration of experiment B increased and then fluctuated prominently. The organic matter content of both experiments reduced rapidly during the mesophilic–thermophilic phase and decreased gradually during the cooling–maturation phase. On day 6, the standard deviation of the organic matter content in experiment B was relatively high, and the results of three duplicates were 65.1%, 65.1% and 78.9%, perhaps because the composting samples were not homogeneous. As shown in Fig. 1B, the soluble sugar content of experiment B was initially higher than that of experiment A, but from Day 4 onwards, it fell significantly lower than that of experiment A (*P *<* *0.05). As this work focuses on the microbial kinetics during composting, the variation in chemical parameters such as chemical oxygen demand (COD) and total organic carbon (TOC) has not been included. Also as a fundamental indicator reflecting the oxygen demand of microorganisms, oxygen uptake rate (OUR) has been investigated in our previous studies (Ge *et al*., 2015, 2016a,2016b). Some composting research found that the trends of COD and OUR were similar and that both exhibited oscillations (Kalamdhad and Kazmi, 2009; Singh *et al*., 2016), while TOC usually decreased with time (Grigatti *et al*., 2011; Hait and Tare, 2011). However, the relationship between *COD*,* TOC* and bacterial kinetics will be addressed in our future work.

### Optimization of PMA‐treatment conditions for quantification of *X*_viable_ of pig manure–wheat straw composting materials

**Figure 2:**
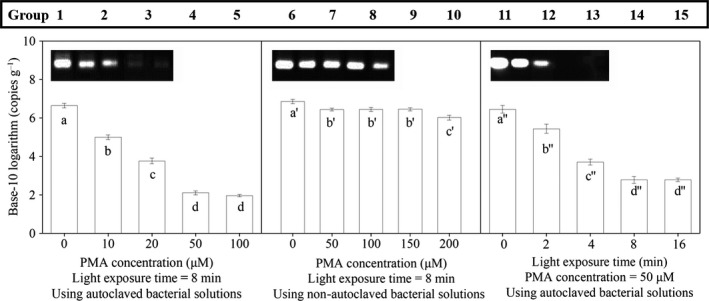
Electrophoretograms and qPCR results of groups 1–15. *Note*: The gene copy number was log 10‐transformed to meet normality assumptions in the statistical analysis; the columns in the same panel marked by the same letter are not significantly different at a significance level of 0.05; error bars represent the standard deviation of triplicate measurements.

The electrophoretograms and qPCR results of groups 1–15 are shown in Fig. 2, where the gene copy number was log 10‐transformed to meet normality assumptions in statistical analysis.

The results for the bacterial solutions in groups 1–5 are shown in the first panel of Fig. 2. These five groups were autoclaved and then treated with different PMA concentrations to determine the optimal PMA concentration. Group 1 without PMA showed a bright electrophoretic band and the highest gene copy number, which was taken to represent the level of DNA amplification from viable bacteria and decayed bacteria together. As the PMA concentration increased from 0 to 50 μM (groups 1 to 4), the brightness of the band faded, and the gene copy number decreased significantly (*P *<* *0.05), implying that the inhibition of amplification of the DNA of decayed significantly increased with the increasing PMA concentration. When the autoclaved bacterial solutions were subjected to a PMA concentration of 50 μM or higher (groups 4 and 5), the electrophoretic band disappeared, and the gene copy number was extremely low, indicating an almost complete prevention of amplification of the DNA of the decayed bacteria. However, the DNA of some decayed bacteria could be still amplified, probably because these decayed bacteria retained intact cytomembranes that PMA could not permeate (Nocker *et al*., 2006). According to the statistical results, the gene copy number did not change significantly as the PMA concentration was raised from 50 to 100 μM (*P *>* *0.05). Therefore, 50 μM was considered the minimum PMA concentration inhibiting qPCR amplification of the DNA of decayed bacteria and used for groups 11–16. The value is higher than the minimum PMA concentration (30 μM) for the pure bacterial strain (Nocker *et al*., 2006), which could be explained by a higher degree of turbidity in the solutions containing pig manure (van Frankenhuyzen *et al*., 2013).

Groups 6–10 were non‐autoclaved bacterial solutions treated with different PMA concentrations. The middle panel of Fig. 2 shows that, as the PMA concentration increased from 0 to 50 μM (groups 6 and 7), there was a significant decrease in the gene copy number (*P *<* *0.05), indicating that PMA effectively bound the exposed DNA of decayed bacteria, and thereby hindered the amplification of the DNA of the decayed bacteria. Therefore, the decrease in the gene copy number from group 6 to group 7 should be attributed to the decayed bacteria in the non‐autoclaved solutions. With an increase in PMA concentration from 50 to 150 μM (groups 7–9), the gene copy number did not change significantly (*P *>* *0.05), implying similar performance of PMA at both levels of concentration. As the PMA concentration increased from 150 to 200 μM (groups 9 and 10), the gene copy number fell by a statistically significant amount (*P *<* *0.05), which suggested that a PMA concentration as high as 200 μM exerted toxicity towards the viable bacteria, producing a mortality rate of 63%. The toxicity could be explained by the infiltration of excessive PMA to the viable cells (Gyawali *et al*., 2016). Therefore, it is necessary to determine the maximum PMA concentration for specific samples, especially for manure, sludge and soil (Gyawali *et al*., 2016). From the above, 150 μM was taken as the maximum PMA concentration, which would not inhibit qPCR amplification of the DNA of viable bacteria. The result is higher than the maximum PMA concentration reported for *Escherichia coli* and *Salmonella* (100 and 80 μM, respectively) (Luo *et al*., 2010; Yu *et al*., 2015), possibly because of the higher degree of turbidity in the solutions in this study.

In groups 11–15, the research objective was to find the effect time of light exposure on autoclaved bacterial solutions. Comparison between groups 11 and 12 showed that the brightness of the band and the copy number decreased significantly (*P *<* *0.05) after a 2 min light exposure, indicating that light exposure was a necessity for the effective application of PMA. As the light exposure time was increased to 8 min (group 14), the weakness of the electrophoretic band and the extremely low copy number demonstrated a notable inhibition of amplification of the DNA of decayed bacteria. There was no noted improvement when the light exposure time was extended from 8 to 16 min (groups 14 and 15), so 8 min was chosen as the minimum light exposure time inhibiting qPCR amplification of the DNA of decayed bacteria. Previous studies showed that the optimal light exposure time was only 1 min with a light power of 750 W for *Enterobacter sakazakii* (Cawthorn and Witthuhn, 2008) and more than 10 min with a light power of 500 W for activated sludge (van Frankenhuyzen *et al*., 2013; Guo and Zhang, 2014), whereas it was 8 min with a light power of 650 W in this study. The discrepancy between these studies and our results implied that the light exposure time might be inversely proportional to the light power. Moreover, with the same treatment conditions, group 14 showed a gene copy number similar to that of group 4, which demonstrates a good reproducibility of the PMA–qPCR results.

As shown in Fig. 2, the DNA of some decayed bacteria in autoclaved groups 4, 5, 14 and 15 was still amplified. There might be two reasons for this. First, the autoclave procedure used in this study belongs to the class of rapid sterilization methods, and under such methods, the morphology of cells may remain intact and the cells remain impermeable to PMA (Trevors, 2012; Lawn and Nicol, 2015). Second, a small number of bacteria may enter a viable‐but‐non‐culturable (VBNC) state as a response to unfavourable conditions occurring during autoclaving. In this case, the cells cannot grow but retain their cytomembranes (Ferrentino *et al*., 2015). However, the statistical analysis of the data as shown in Fig. 2 indicates that the decayed bacteria with intact cytomembranes accounted for only 0.002–0.003% of the total decayed bacteria. In addition, composting processes would not have the effects of rapid pasteurization procedures such as autoclaving. Therefore, the decayed bacteria with intact morphology were not considered in the calculation of the kinetic parameters during the composting operation.

The above results indicated that, to effectively discern the viable and decayed bacteria of the samples of manure composting, the PMA concentration and light exposure time should be in the range of 50–150 μM and no <8 min respectively. Given the high turbidity of the solutions, 100 μM was accepted as the optimal PMA concentration, which is in accordance with the results for activated sludge and wastewater (Guo and Zhang, 2014; Li *et al*., 2014), possibly because of the similar physicochemical properties of the solutions. According to the results from groups 11–15, a period of 8 min was chosen as the optimal light exposure time.

### Quantification results and related mechanisms of bacterial growth and decay during pig manure–wheat straw aerobic composting

**Figure 3:**
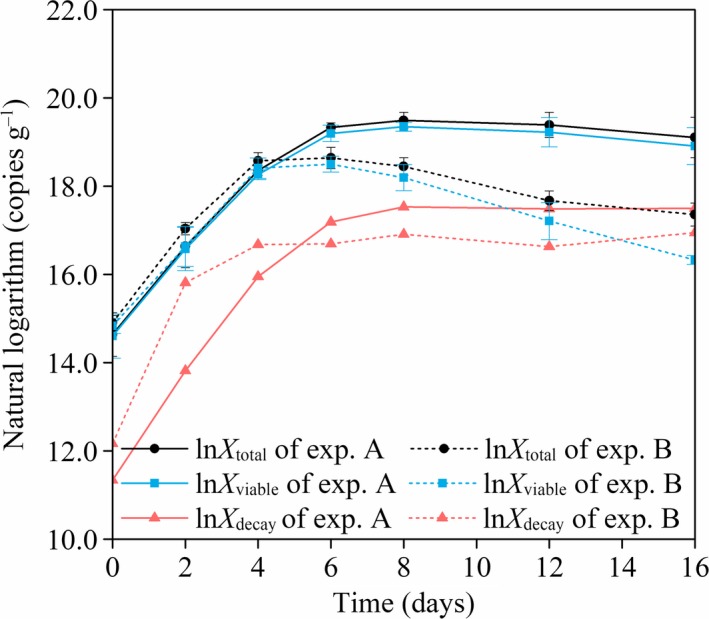
Variation of *X*_total_, *X*_viable_ and *X*_decay_ during pig manure–wheat straw aerobic composting. *Note*:* X*_total_, *X*_viable_ and *X*_decay_ are the concentration of total, viable and decayed bacteria respectively; *X*_total_, *X*_viable_ and *X*_decay_ were natural log‐transformed to meet normality assumptions in the statistical analysis; exp represents experiment; error bars represent the standard deviation of triplicate measurements.

To meet normality assumptions in statistical analysis, the variables *X*_total_, *X*_viable_ and *X*_decay_ were natural log‐transformed. Based on the qPCR and optimized PMA–qPCR methods, the variation of ln*X*_total_, ln*X*_viable_ and ln*X*_decay_ during pig manure–wheat straw aerobic composting and statistical analysis results is illustrated in Fig. 3 and Table 2.

**Table 2:** Quantification and statistical analysis results of *X*_total_, *X*_viable_ and *X*_decay_ during pig manure–wheat straw aerobic composting

Experiment	Concentration or proportion	Composting progress						
		Day 0	Day 2	Day 4	Day 6	Day 8	Day 12	Day 16
A	ln*X*_total_ (copies g^−1^)	14.6 ± 0.5^aA^	16.6 ± 0.5^bA^	18.4 ± 0.1^cA^	19.3 ± 0.1^dA^	19.5 ± 0.2^dA^	19.4 ± 0.3^dA^	19.1 ± 0.5^dA^
	ln*X*_viable_ (copies g^−1^)	14.6 ± 0.5^aC^	16.6 ± 0.5^bC^	18.3 ± 0.1^cC^	19.2 ± 0.2^dC^	19.4 ± 0.1^dC^	19.2 ± 0.3^dC^	18.9 ± 0.4^dC^
	ln*X*_decay_ (copies g^−1^)	11.3	13.8	16.0	17.2	17.5	17.5	17.5
	*X*_viable_/*X*_total_ (%)	96.6	94.4	91.0	88.3	86.1	85.5	81.2
	*X*_decay_/*X*_total_ (%)	3.4	5.6	9.0	11.7	13.9	14.5	18.8
B	ln*X*_total_ (copies g^−1^)	14.9 ± 0.2^aA^	17.0 ± 0.1^bA^	18.6 ± 0.2^cA^	18.6 ± 0.2^cB^	18.5 ± 0.2^cB^	17.7 ± 0.2 ^dB^	17.4 ± 0.3^bdB^
	ln*X*_viable_ (copies g^−1^)	14.8 ± 0.2^aC^	16.6 ± 0.5^bC^	18.4 ± 0.2^cC^	18.5 ± 0.2^cD^	18.2 ± 0.3^cD^	17.2 ± 0.4^dD^	16.3 ± 0.1^bD^
	ln*X*_decay_ (copies g^−1^)	12.2	15.8	16.7	16.7	16.9	16.6	17.0
	*X*_viable_/*X*_total_ (%)	93.6	70.9	85.2	86.0	78.8	65.4	35.1
	*X*_decay_/*X*_total_ (%)	6.4	29.1	14.8	14.0	21.2	34.6	64.9

*X*_total_, *X*_viable_ and *X*_decay_ are the concentration of total, viable and decayed bacteria respectively; *X*_decay_ =* X*_total_ − *X*_viable_; *X*_total_, *X*_viable_ and *X*_decay_ were natural log‐transformed to meet normality assumptions in the statistical analysis; values are expressed as the mean ± standard deviation (*n *=* *3); ^a,b,c,d^significant difference of ln*X*_total_ (or ln*X*_viable_) between different time points in the same row; ^A,B^significant difference of ln*X*_total_ between experiments A and B at each time point; ^C,D^significant difference of ln*X*_viable_ between experiments A and B at each time point; the significance level is 0.05.

It could be seen that viable and decayed bacteria coexisted in the whole composting period. As the experiments approached an end, the proportion of viable bacteria (*X*_viable_/*X*_total_) in experiment B fell as low as 35.1%. Such large changes in the course of a process run imply that it is important to be able to quantify *X*_viable_ for composting processes.

As shown in Fig. 3 and Table 2, the variation of the bacterial concentration exhibited some similarities and some differences between experiments A and B. During the mesophilic phase, the value of ln*X*_total_ (or ln*X*_viable_) showed no difference between experiments A and B (*P *>* *0.05), ln*X*_viable_ increased linearly, and ln*X*_decay_ increased correspondingly. This indicated a logarithmic‐growth stage synchronizing with a rapid‐decay stage. From the middle of the thermophilic phase (around day 5), the oxygen concentration of both experiments started to increase (Fig. 1), which implied that the bacterial metabolism had begun to slow down and the bacterial growth and decay in both experiments entered into a stable stage at the same time point. However, after this time point, the variation of the bacterial concentration between experiments A and B deviated significantly. In experiment A, ln*X*_viable_ remained almost unchanged (*P *>* *0.05) from the middle of the thermophilic phase to the maturation phase, indicating that the bacterial growth in experiment A remained at the stable stage until the end. In experiment B, however, ln*X*_viable_ was quite constant (*P *>* *0.05) during days 6–8, implying that the bacterial growth in experiment B was at the stable stage for that time period. But during days 8–16, ln*X*_total_ and ln*X*viable in experiment B decreased significantly (*P *<* *0.05) while ln*X*_decay_ remained almost unchanged. The decrease in *X*_total_ agrees with the studies of poultry manure, feather waste and sewage sludge composting (Chroni *et al*., 2009; Korniłłowicz‐Kowalska and Bohacz, 2010; Pepe *et al*., 2013). The decreasing *X*_viable_ and constant *X*_decay_ in experiment B perhaps implied that bacteria were decomposing by means of autolysis and autophagy, in which case the bacterial decay was occurring at a decomposition stage. A possible explanation for the autolysis and autophagy of bacteria in experiment B is that, with continued degradation of organic matter and near‐exhaustion of soluble sugar in the composting materials (Fig. 1), the bacteria may have been forced to consume their own organic matter. As previous composting studies did not measure the variation in viable and decayed bacteria, there is no available report on the bacterial autolysis and autophagy occurring in composting. However, some studies on the anaerobic digestion of pig manure (Harvey *et al*., 1984) and sludge (Alphenaar *et al*., 1993; Yang *et al*., 2010) have demonstrated the phenomenon of bacterial autolysis and autophagy during the latter phase of digestion. Therefore, we conjecture that the significant decrease in ln*X*_viable_ in experiment B was caused by bacterial autolysis and autophagy. Regarding the duration of the stable stage of bacterial growth as shown in Fig. 3, experiment B had a shorter stable stage than experiment A. This was probably due to the fact that the soluble sugar content of experiment B dropped dramatically from Day 4 onward and remained at a low level during days 8–16 (Fig. 1B), which might be insufficient to support the metabolism of viable bacteria during days 8–16. It could be inferred that the duration of the stable stage of bacterial growth might be related to the soluble sugar content of the composting materials; plentiful organic matter may result in an extension of composting. As this observation is interesting, the effects of different organic compounds such as soluble sugar, lignocellulose, proteins and fats on the bacterial kinetics will be further investigated in our future work.

In existing studies of aerobic composting, the bacterial decay rate was proportional to the growth rate when there was sufficient soluble substrate for bacterial metabolism (Angelidaki *et al*., 1993; Oudart *et al*., 2015). Hence, the decay rate was positively correlated with the growth rate. Therefore, during the mesophilic–thermophilic phase, ln*X*_decay_ increased with the increase of ln*X*_viable_ (see Fig. 3). However, when the composting process entered the cooling phase, the bacterial growth and decay entered a stable state, perhaps because the soluble substrate became insufficient for bacterial proliferation (Haug, 1993).

To summarize the above quantification results, during the mesophilic–early thermophilic phase, bacteria were at the logarithmic‐growth stage that synchronized with the rapid‐decay stage; from the middle of the thermophilic phase, bacterial growth entered into the stable stage, and possibly further entered into the decomposition phase; the duration of the stable stage of bacterial growth was positively related to the soluble sugar content of the composting materials. Quantification of *X*_total_, *X*_viable_ and *X*_decay_ enables the identification of the logarithmic‐growth, stable and decomposition stages of bacterial growth and thereby enables better prediction of the variation of composting temperature and the degree of maturation of the composting materials. A better understanding of these details of the composting process will ultimately enable researchers to improve composting efficiency.

### Determination results of *μ* and *k*_d_ for pig manure–wheat straw aerobic composting

During the mesophilic–early thermophilic phase, the variation of *X*_viable_ and *X*_decay_ of both experiments followed the Monod equations, i.e., Eqs. (1) and (2). Given no significant differences of ln*X*_total_ (or ln*X*_viable_) between experiments A and B (Table 2), the mean value of *X*_total_ (or *X*_viable_) of the two experiments was simulated using Eqs. (1) and (2) to calculate *μ* and *k*_d_. The values of *μ* and *k*_d_ were 0.0317 ± 0.0033 h^−1^ and 0.0019 ± 0.0000 h^−1^ respectively (the values of *R* ^2^ for both equations were 1.00).

The values of *μ* and *k*_d_ reported in the present study and some previous studies are listed in Table 3. The comparison as shown in Table 3 indicates that the value of *μ* (or *k*_d_) differs significantly across different composting materials. Among these studies, Kaiser (1996), Stombaugh and Nokes (1996), Seki (2000) and Vidriales‐Escobar *et al*. (2017) incorporated a temperature correction factor [*f*(*T*)] into the calculation of the corrected specific growth rate [*μ*(*T*)], which varied with the composting temperature (*T*). The average values of *μ*(*T*) in the above studies were 0.07, 0.29, 0.02 and 0.05 h^−1^. The value obtained for *μ* in the present study based on the quantification results of *X*_viable_ and *X*_decay_ is *μ *= 0.0317 ± 0.0033 h^−1^, which approximates the values in the early research using similar composting materials, such as chicken manure–rice bran–sawdust (Seki, 2000) and pig manure–wheat straw (Wang and Witarsa, 2016). As for *k*_d_, the range of *k*_d_ in the previous studies, as shown in Table 3, was (0.01–0.25) times the value of *μ*, while some past research assumed *k*_d_ = 0.05 *μ* (Angelidaki *et al*., 1993; Oudart *et al*., 2015). The *k*_d_ obtained in the present study is (0.06 ± 0.01) times the value of *μ*, which falls within the published range.

**Table 3:** Specific growth rate coefficient (*μ*) and decay rate coefficient (*k*_d_) reported in this study and previous studies

Composting materials	Scale	Determination method of *μ* and *k*_d_	Initial OM (%)	*μ* (h^−1^)	*k*_d_ (h^−1^)	Simulation accuracy	References
Pig manure–wheat straw	16 l reactor	Variation of the concentration of viable and decayed bacteria	81.3	0.03	0.002, 0.06 *μ*	*R* ^2^ * *=* *0.93	This study
Horticultural residues–straw	0.7 m^3^ reactor	Relationship between the variation of bacterial concentration and composting temperature	/	0.07^a^	0.001, 0.01 *μ*	*R* ^2^ * *=* *0.80	Kaiser (1996)
Corncobs	2.1 l reactor	Relationship between the variation of bacterial concentration and composting temperature	/	0.29^a^	0.025, 0.13 *μ*	/	Stombaugh and Nokes (1996)
Chicken manure–rice bran–sawdust	18.8 l reactor	Relationship between the variation of bacterial concentration and composting temperature	75	0.02^a^	0.001, 0.25 *μ*	*R* ^2^ * *=* *0.78	Seki (2000)
Poultry manure–barley straw	12.3 l reactor	Relationship between the variation of bacterial concentration and ammonia emission rate	/	0.60	0.025, 0.04 *μ*	*R* ^2^ * *=* *0.86	Liang *et al*. (2004)
Fruit pulp–cattle manure–sludge	15 l reactor	Relationship between the variation of bacterial concentration and dry matter content	/	0.20	0.030, 0.15 *μ*	MD* *=* *2.14%	Sole‐Mauri *et al*. (2007)
Pig slurry–wheat straw	8 m^3^ pile	Relationship between the variation of bacterial concentration and nitrogen content	15.8–29.1	0.20	0.008, 0.04 *μ*	/	Oudart *et al*. (2015)
Pig manure–wheat straw	50 l reactor	Relationship between the variation of bacterial concentration and pile density	86.1	0.02	0.002, 0.12 *μ*	*R* ^2^ * *=* *0.99	Wang and Witarsa (2016)
Tobacco industry solid waste	25 l reactor	Relationship between the variation of bacterial concentration and composting temperature	/	0.05^a^	0.036, 0.20 *μ*	*R* ^2^ * *=* *0.92	Vidriales‐Escobar *et al*. (2017)

MD is the mean deviation; OM is the organic matter content; *R* ^2^ is the coefficient of determination; *T* is the composting temperature.

**a**. The average of the corrected specific growth rate coefficient [*μ*(*T*)].

In previous studies in which the PMA–qPCR method was not employed, separate measures of *X*viable and *X*_decay_ could not be obtained. Hence, when calculating *μ*, the *X*viable in Eq. 1 was replaced with *X*_total_, and *k*_d_ was considered to be negligible or assumed to be equal to 0.05 *μ* (Angelidaki *et al*., 1993; Oudart *et al*., 2015). For comparison, the present study also evaluated *μ* by replacing *X*_viable_ with *X*_total_ using the data set of this study: When *k*_d_ was neglected, the measured value for *μ *= 0.0166 ± 0.0093 h^−1^; when *k*_d_ = 0.05 *μ*,* μ *= 0.0176 ± 0.0107 h^−1^. However, both of the above *μ* values were lower than the result obtained from the PMA–qPCR method, i.e., *μ *= 0.0317 ± 0.0033 h^−1^. Therefore, the *μ* value might be underestimated the relative concentration of viable and decayed bacteria is not considered, because this causes the degree of degradation of organic matter to be underestimated. Since an understanding of the bacterial kinetics of the process is important for simulating the degradation of organic matter and the emission of greenhouse gases during aerobic composting, the improvement in parameter estimates made possible by the new method may enable researchers to understand these mechanisms better.

Existing studies indicate that the degradation of organic matter during aerobic composting follows the first‐order equation (Haug, 1993):

(3) $$\text{OM}={\text{OM}}_{0}\cdot e^{-\mathrm{kt}}$$

where OM is the organic matter content (%); OM_0_ is the initial organic matter content (%); *k* is the degradation rate constant (h^−1^); *t* is the composting time (h). By fitting the variation of OM in our study across time *t*, the *k* value was estimated to be 0.001 ± 0.000 h^−1^, which was 0.03 ± 0.01 times the value of *μ*. Obviously, *k * <  *μ*, as it should be, since there is a bacterial yield coefficient (*Y*_X_) (which captures the interplay between bacterial growth and organic matter degradation), which represents the bacterial yield generated by consuming one unit mass of organic matter (Vidriales‐Escobar *et al*., 2017; Rentería‐Tamayo *et al*., 2018). A study on Chinese hamster ovary cells implied that *μ *= 0.005 h^−1^, while *k* varied with different organic compounds and was in a range of 0.002–0.004 h^−1^, i.e., *k *= (0.06–0.66)*μ* (Kurano *et al*., 1990). A study of the aerobic degradation of sludge reported that the *μ* and *k* of bacteria were 0.048 and 0.006 h^−1^, respectively, which implies a value of *k *=* *0.13 *μ* (Accashian *et al*., 1998). Lastly, a study of the aerobic degradation of groundwater which involved a variety of different organic compounds found that *k *= (0.03–0.21)*μ* and found that *k* varied with different organic compounds – the more difficult the organic compound to be degraded, the lower the *k* value, and the lower the ratio of *k* to *μ* (Powers *et al*., 2001).

Especially for developing countries such as China, Vietnam and India, which feature massive solid wastes but limited land for waste management, there is a tremendous demand for automatic and intelligent composting strategies. With the help of the PMA–qPCR method, operators of composting facilities could perform real‐time monitoring of *X*_viable_ and *X*_decay_ and thereby calculate the kinetic parameters of ongoing processes more precisely. They would be informed of variations in the quantities of various bacteria, including pathogens, and would be better able to estimate the degree of degradation of organic matter and the maturity of the compost. Consequently, operators could optimize composting procedures to facilitate microbial growth, accelerate decomposition of wastes and produce high‐quality compost free of harmful bacteria.

## Conclusions

As an understanding of the kinetics of bacterial growth and decay is important for simulating the degradation of organic matter and emissions of greenhouse gases during aerobic composting, improved estimates of *μ* and *k*_d_ would help to better understand the above mechanisms and estimate other parameters involved. In order to characterize the parameters, a PMA–qPCR method was applied, and the PMA‐treatment conditions were optimized for pig manure–wheat straw aerobic composting, including a PMA concentration of 100 μM and an exposure time of 8 min. The resulting variation of *X*_total_, *X*_viable_ and *X*_decay_ showed that during the mesophilic–early thermophilic phase, bacteria were at a logarithmic‐growth stage that synchronized with a rapid‐decay stage. During the late thermophilic–maturation phase, bacterial growth either remained at a stable stage or entered a decomposition stage, which might be related to a possible decline in the soluble sugar content of the composting materials. The *μ* value obtained in studies which did not employ the PMA–qPCR method was lower than the result obtained from the PMA–qPCR method, suggesting that there is a danger of underestimating the extent of bacterial growth if we fail to consider the concentrations of viable and decayed bacteria separately, due to an underestimation of the degree of degradation of organic matter.

## Experimental procedures

### Aerobic composting experiments and physicochemical analysis

Composting materials consisted of pig manure (10.0 kg) and chopped wheat straw (1.0 kg), which were collected from the livestock and poultry test site of the Chinese Academy of Agricultural Sciences (Changping, Beijing, China) and suburban areas of Beijing respectively. During manual mixing, 1.0 kg of deionized water was added to achieve a moisture content of 50–67% (Haug, 1993). The materials were mixed thoroughly and divided into two equal portions to be loaded into each of two 15‐l cylindrical reactors (0.40 m height × 0.25 m inside diameter) (Lü *et al*., 2008). We established two independent aerobic composting experiments (A and B). Each of them was 16 days in duration. The ventilation pattern and aeration rate were set to 1 h on/1 h off and 0.35 l min^−1^ based on previous work (Ge *et al*., 2015). Composting temperature and oxygen concentration in the upper part of the reactor were determined using a thermocouple (Pt100, Thermocoax, Hamburg, Germany) located in the middle of the composting mixture and an oxygen sensor (O2S‐FR‐T2‐18X; Apollo Electronics Co., Ltd., Zhuhai, China) respectively (Ge *et al*., 2016a,2016b). Samples were collected on days 0, 2, 4, 6, 8, 12 and 16. Each time, an amount of approximately 200 g of sample was taken from each reactor. Not <50 g was taken out and stored at −20°C for qPCR and PMA–qPCR analysis, while the remaining amount was stored at 4°C for physicochemical analysis.

The moisture content and organic matter content of the samples were measured according to Test Methods for the Examination of Composting and Compost (TMECC) standard procedures (03.09‐A and 05.07‐A) (U.S. Composting Council, 2002). The carbon to nitrogen ratio was calculated from total carbon and total nitrogen, which were determined by dry combustion using an elemental analyser (Vario MACRO, Elementar, Hanau, Germany). The soluble sugar content was determined using a spectrophotometer (UV‐2550, Shimadzu, Tokyo, Japan) according to the anthrone–sulphuric acid method (Yemm and Willis, 1954). The above physicochemical properties were determined using three replicates.

The physicochemical properties of the initial composting mixtures of experiments A and B are illustrated in Table 4. The initial values of all the physicochemical properties conformed to the requirements of composting standards (Haug, 1993). The statistical analysis showed that the initial organic matter content of experiment A was significantly higher than that of experiment B.

**Table 4:** Physicochemical properties of the initial composting mixtures of experiments A and B

Physicochemical properties	Experiment A	Experiment B	*F* value	*P* value
Moisture content (%)^a^	56.2 ± 2.6	56.5 ± 2.8	0.0	0.9
Organic matter content (%)^b^	83.3 ± 1.4	79.3 ± 1.2	14.6	0.0
Total carbon content (%)^b^	42.0 ± 0.2	43.9 ± 2.2	2.2	0.2
Total nitrogen content (%)^b^	2.6 ± 0.2	2.8 ± 0.1	3.0	0.2
Carbon to nitrogen ratio^b^	16.6 ± 1.4	15.8 ± 1.0	0.6	0.5

Values are expressed as the mean ± standard deviation (*n *=* *3).

**a**. Measurements based on wet weight.

**b**. Measurements based on dry weight; *F* and *P* values were derived from one‐way ANOVA.

### qPCR analysis and quantification of *X*_total_ of pig manure–wheat straw composting materials

An aliquot of 5 g of a composting sample was added to 50 ml of sterile water and mixed completely to generate a bacterial solution. The DNA of the solution was extracted using a TIANamp Bacteria DNA Kit (Tiangen Biotech Beijing Co., Ltd., China) followed by amplification on a real‐time PCR system (ABI 7500; Applied Biosystems, Foster City, CA, USA) with a forward primer (519F, 5‐CAGCMGCCGCGGTAATWC‐3), and reverse primer (907R, 5‐CCGTCAATTCMTTTRAGTTT‐3) (Zhang *et al*., 2015). The qPCR products were electrophoresed on a 1% agarose gel containing ethidium bromide and visualized using a gel image processing system (Tanon‐1600; Tanon Science & Technology Co., Ltd., Shanghai, China). The qPCR products were then cloned using a pUC‐T TA cloning kit (CoWin Biosciences, Beijing, China). The products were transformed into *E. coli* DH5α competent cells. The transformants were incubated in broth. Positive clones were selected and further identified by qPCR using the primers. The plasmids of the positive clones were saved as plasmid DNA standards. Serial 10‐fold dilutions of the plasmid standards were prepared (Selvam *et al*., 2012). Calibration curves were generated with Sequence Detection System software (version 2.0; Applied Biosystems) according to the qPCR results of the plasmid DNA standards and dilutions.

An aliquot of 5 g of each sample from experiments A and B was added to 50 ml sterile water and mixed completely to generate bacterial solutions. The DNA of the solutions were extracted and tested by qPCR as described as above to obtain *X*_total_. Each sample was tested in triplicate.

### Optimization of PMA‐treatment conditions

An aliquot of 5 g of a composting sample was added to 50 ml of sterile water and mixed completely to generate a bacterial solution. An aliquot of 20 ml of the bacterial solution was autoclaved at 121°C for 30 min to generate an autoclaved solution. Based on the results of preliminary experiments, PMA treatment contained 15 groups; each group was generated in triplicate; the design is listed in Table 5. Groups 1–5, 6–10 and 11–15 were used to identify the minimum PMA concentration inhibiting qPCR amplification of the DNA of decayed bacteria, the maximum PMA concentration not inhibiting qPCR amplification of the DNA of viable bacteria, and the minimum light exposure time inhibiting qPCR amplification of the DNA of decayed bacteria respectively. In groups 1–5, the autoclaved solution, PMA stock solution (Biotium, Hayward, CA, USA) and sterile water were mixed to generate five PMA concentrations (0, 10, 20, 50 and 100 μM). The tubes were wrapped in aluminium foil, fixed onto an ice box and placed on a shaker (SK‐O180‐E; DRAGON‐LAB, Beijing, China) for 10 min (Elizaquível *et al*., 2014; Guo and Zhang, 2014; Li *et al*., 2014). Then, the aluminium foil was removed, and the tubes were exposed to the light from a 650‐W halogen lamp (64540, Osram, Munich, Germany) for 8 min to photoactivate the exposed DNA of the decayed bacteria. The tubes were gently shaken during the exposure. In groups 6–10, the non‐autoclaved solution, PMA stock solution and sterile water were mixed to generate five PMA concentrations (0, 50, 100, 150 and 200 μM). The light exposure process was the same as described above. In groups 11–15, autoclaved solution, PMA stock solution and sterile water were mixed to generate a PMA concentration of 50 μM which was determined according to the results of the groups 1–5. Light exposure time was set as 0, 2, 4, 8 and 16 min. The DNA of the PMA‐treated solutions was extracted and examined by qPCR using the same procedures as described as above. The results were used to identify the best PMA‐treatment conditions.

**Table 5:** Design of PMA treatment

Technical parameter	Group	Bacterial solution, 100 μl	PMA concentration (μM)	Light exposure time (min)
Minimum PMA concentration inhibiting qPCR amplification of DNA of decayed bacteria	1	Autoclaved	0	8
	2	Autoclaved	10	8
	3	Autoclaved	20	8
	4	Autoclaved	50	8
	5	Autoclaved	100	8
Maximum PMA concentration not inhibiting qPCR amplification of the DNA of viable bacteria	6	Non‐autoclaved	0	8
	7	Non‐autoclaved	50	8
	8	Non‐autoclaved	100	8
	9	Non‐autoclaved	150	8
	10	Non‐autoclaved	200	8
Minimum light exposure time inhibiting qPCR amplification of the DNA of decayed bacteria	11	Autoclaved	50	0
	12	Autoclaved	50	2
	13	Autoclaved	50	4
	14	Autoclaved	50	8
	15	Autoclaved	50	16

### Quantification of *X*_viable_ and *X*_decay_ of pig manure–wheat straw composting materials by the optimized PMA–qPCR method

The bacterial solutions of all samples from experiments A and B were treated by the optimized PMA‐treatment conditions. Each sample was treated in triplicate. The DNA of the PMA‐treated solutions was extracted and tested by qPCR using the same procedures as described as above to acquire *X*_viable_. The difference between *X*_total_ and *X*_viable_ was noted as *X*_decay_.

### Determination of *μ* and *k*_d_ for pig manure–wheat straw aerobic composting

The variation of *X*_total_ and *X*_viable_ was fitted by Eqs. (1) and (2) to calculate *μ* and *k*_d_ by a universal global optimization algorithm using mathematical software (1stOpt; 7D‐Soft High Technology Inc., Beijing, China). The fit quality was evaluated by the determination coefficient (*R* ^2^).

### Statistical analysis

One‐way analysis of variance and least significant difference tests were performed using statistical software (SPSS 15.0; SPSS Inc., Chicago, IL, USA), with the significance level set at *P *=* *0.05, to determine whether there was any significant difference in (i) initial physicochemical properties between experiments A and B; (ii) the gene copy number between groups 1–5, 6–10 and 11–15; (iii) the bacterial concentration between different time points; (4iv) the bacterial concentration between experiments A and B.

## Conflicts of interest

None declared.
